# GLP-1 and GLP-2 Orchestrate Intestine Integrity, Gut Microbiota, and Immune System Crosstalk

**DOI:** 10.3390/microorganisms10102061

**Published:** 2022-10-19

**Authors:** Nyan Abdalqadir, Khosrow Adeli

**Affiliations:** 1 Molecular Medicine, Research Institute, The Hospital for Sick Children, Toronto, ON M5G 1H3, Canada; 2 Department of Laboratory Medicine and Pathobiology, University of Toronto, Toronto, ON M5S 1A8, Canada; 3 Department of Biology, College of Science, University of Sulaimani, Sulaymaniyah 46001, Iraq; 4 Department of Biochemistry, University of Toronto, Toronto, ON M5S 1A8, Canada; 5 Department of Physiology, University of Toronto, Toronto, ON M5S 1A8, Canada

**Keywords:** glucagon-like peptide 1 (GLP-1), glucagon-like peptide 2 (GLP-2), gut microbiota, intestinal barrier integrity, inflammation, gut immunity, metabolic syndrome

## Abstract

The intestine represents the body’s largest interface between internal organs and external environments except for its nutrient and fluid absorption functions. It has the ability to sense numerous endogenous and exogenous signals from both apical and basolateral surfaces and respond through endocrine and neuronal signaling to maintain metabolic homeostasis and energy expenditure. The intestine also harbours the largest population of microbes that interact with the host to maintain human health and diseases. Furthermore, the gut is known as the largest endocrine gland, secreting over 100 peptides and other molecules that act as signaling molecules to regulate human nutrition and physiology. Among these gut-derived hormones, glucagon-like peptide 1 (GLP-1) and -2 have received the most attention due to their critical role in intestinal function and food absorption as well as their application as key drug targets. In this review, we highlight the current state of the literature that has brought into light the importance of GLP-1 and GLP-2 in orchestrating intestine–microbiota–immune system crosstalk to maintain intestinal barrier integrity, inflammation, and metabolic homeostasis.

## 1. Introduction

Glucagon-like peptides (GLP-1 and GLP-2) are gut-derived peptides that are secreted in response to ingested nutrient from enteroendocrine L-cells located predominantly in the distal small intestine and colon [1,2]. GLP-1 and GLP-2 exert their actions through binding to distinct G-protein-coupled transmembrane receptor, GLP-1R and GLP-2R, respectively [3,4,5,6]. Depending on the tissues that express these receptors, GLP-1 and GLP-2 elicit different effects. GLP-1R activation is shown to promote glucose metabolism, inhibit gastric emptying, and supress glucagon release [2,7,8,9,10]. Through the gut-brain axis, increasing levels of circulating GLP-1 or pharmacological activation of GLP-1R directly lower food intake and regulate body weight [11]. Although GLP-2 is co-secreted in a 1:1 ratio with GLP-1, its main functions in the intestine are to promote crypt cell proliferation, intestine stem cell expansion, and intestinal growth [12,13,14,15,16].

The gut microbiota has recently received a lot of attention due to its broad and comprehensive influence on various organs and other aspects of host physiology. It is well accepted that the composition of gut microbiota depends on many factors, such as regions of the gastrointestinal tract as well as host genetics, nutritional and health status, dietary habits, and antibiotic use. In recent years, along with the technological advances in systems biology, host-microbiota interplay has become a new promising field for molecular and personalized medicine, pharmacokinetics, biomarker discovery, diagnosis, and therapeutic targets. The implication of gut microbiota in human health and diseases have been extensively studied and reviewed [17,18,19,20]. A body of research has shown that the gut environment is crucial for maintaining host physiology and its disturbance can result in a variety of physiological disorders such as loss of insulin sensitivity, impairment of intestinal barrier function, low-grade inflammation, and lipid accumulation, all of which increase the risk of metabolic diseases [11,18,21,22,23,24,25,26]. Additionally, commensal microorganisms form a mutualistic relationship with the host, which is crucial for immune system development and function through production of a variety of metabolites [20,25,27,28,29]. 

In this review, we highlight the recent research and key concepts on possible interactions between the enteroendocrine system, gut microbiota, and host immunity, as well as the implications of these interactions for human health.

## 2. Receptors and Functions of GLP-1 and -2

**Figure 1:**
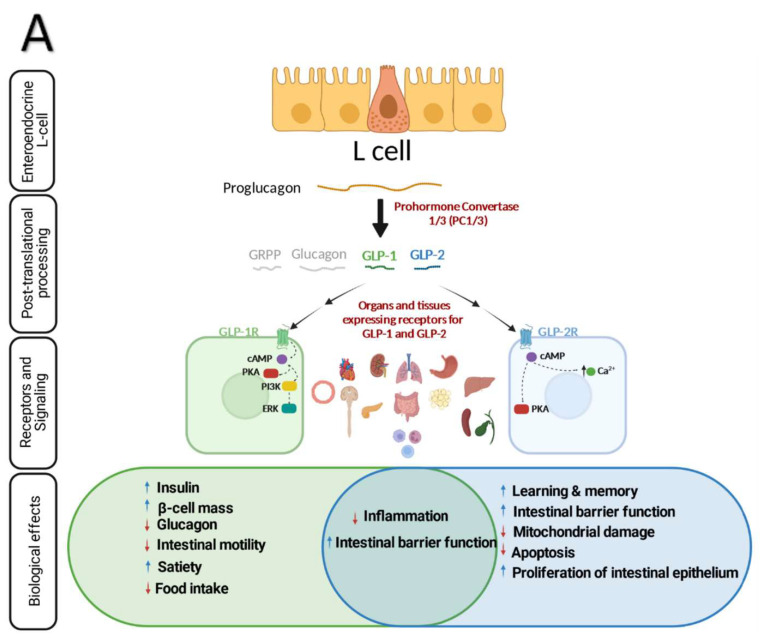

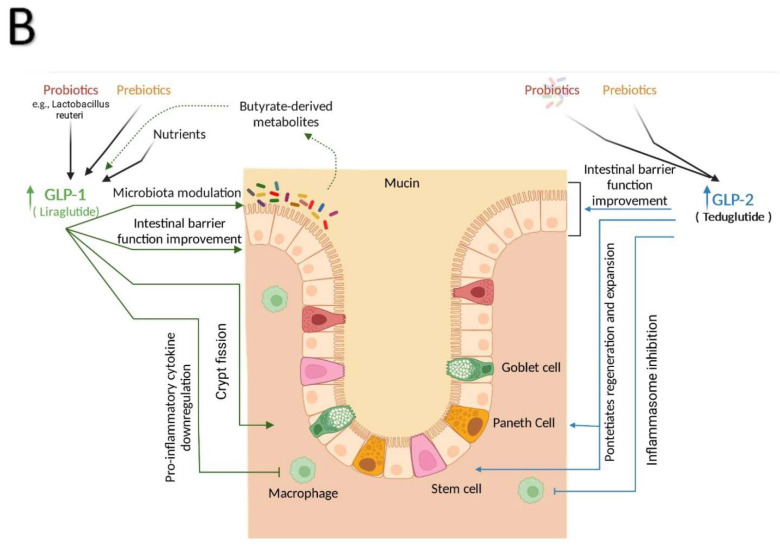
GLP-1 and GLP-2 signaling and the interaction with the gut inflammatory network and gut microbiota. (**A**) GLP-1 and 2 are produced by L-cells via enzyme-mediated cleavage of proglucagon. These gut peptides exert diverse effects through binding to their specific receptors, GLP-1R and GLP-2R, respectively. (**B**) Intestine-specific functions of GLP-1 and -2 are essential to maintain intestine–microbiota–immune system interactions. Created with BioRender.com.

Both GLP-1 and -2 are co-encoded by proglucagon which is a 180-amino acid precursor with five individually processed domains; glicentin-related pancreatic polypeptide (GRPP), glucagon, intervening peptide—1 and 2 (IP-1/2), GLP-1 and GLP-2 [30] and synthesized in pancreatic α cells, intestinal L-cells and neurons located in the hindbrain and hypothalamus [31] (Figure 1A). Tissue specific posttranslational modification of proglucagon produce different active peptides [32]. Prohormone convertase (PC)-2 in the pancreatic α-cells cleaves proglucagon into glucagon, GRPP and a large C-terminal peptide containing both GLPs and IP-2. In intestinal L-cells and specific hindbrain neurons, proglucagon is converted through proconvertase 1/3 L-cell into GLP-1, GLP-2, glicentin, GRPP, oxyntomodulin, and IP-2 [30,33,34,35,36,37,38].

Over the past few decades, the GLP-1 hormone has drawn a lot of interest for its extensive metabolic effects. Dipeptidyl peptidase-4 (DPP-4) inhibitors which increases GLP-1 and gastric inhibitory polypeptide (GIP) incretin hormone concentrations and GLP-1 receptor (GLP-1R) agonists are now widely used to treat type 2 diabetes and obesity [39,40,41]. It has been reported that GLP-1 mediates several beneficial effects on metabolism including maintaining glucose homeostasis by inducing insulin secretion in response to glucose stimulation [1,2], inhibiting glucagon secretion [9] and expanding pancreatic β cell mass [7,42]. GLP-1 also reduces gastric emptying and food intake [1,2,8,10] as well as inhibits intestinal motility [43]. By increasing satiety through central nervous system (CNS) and vagal afferent signaling, GLP-1 can facilitate weight loss. This effect was associated with decreasing cardiometabolic risk factors and inflammatory markers [8,44,45,46]. Along with its possible cardioprotective role, GLP-1 has been implicated in modulating intestinal lipid and lipoprotein metabolism which is perturbed in diabetes. Studies in both rodents and humans showed that activation of GLP-1R or inhibition of DPP-4 resulted in reduction of postprandial chylomicron (CM) secretion [47], decreased postprandial free fatty acid (FFA) levels and triglyceride-rich lipoproteins (TRL) secretion [48,49,50,51]. Moreover, exendin 9-39 mediated inhibition of GLP-1R and genetic ablation of GLP-1R signaling in Glp-1r KO mice increased TRL-apoB48 secretion in vivo [47]. Intracerebroventricular exendin-4 administration markedly reduced the apolipoprotein B48- containing CM levels in hamsters [52]. Recently, Varin et al., reported that sitagliptin mediated gut-specific and systemic inhibition of DPP-4 improved lipid tolerance and TG production in young mice while these effects were not reproduced in old mice [53]. Furthermore, systemic inhibition of DPP-4 activity reduced circulating apoB48 levels and this was accompanied by decreased intestinal CM secretion, conversely; gut selective DPP4 inhibition failed to decrease plasma apoB48 levels [53]. Given that enterocytes are the site for CM synthesis and secretion but do not express GLP-1R [3,54], the effect of GLP 1R agonist on modulation of CM secretion and apoB48 levels is likely the result of indirect mechanisms. Recently, Nahmias. et al., evaluated CM secretion directly using a lymph cannulated rat model to test the impact of physiological GLP-1 levels on lymph flow [55]. The researchers showed that GLP-1R antagonist significantly increased lymph TG concentration independently of gastric emptying as well as increased levels of ApoB48 [55]. This is in line with a study that discovered significant decreases in TRL-TG after six hours of intraperitoneal exendin-4 injection, but no significant changes in apoB48 in fed-state hamsters [52].

GLP-2 was initially characterized as an intestinotrophic hormone secreted by L-cells residing within the epithelium of the small and large intestine. GLP-2 is a short peptide, 33 amino acids, produced as a result of prohormone convertase 1/3-mediated posttranslational processing of proglucagon in intestine and brain. The mechanisms of action and the extent of biological effects of GLP-2 remained undetermined until its action on gut growth was validated using synthetic GLP-2 [56]. Since then, GLP-2 based research gained more attention as a promising therapeutic target of inflammatory and short bowel syndrome [2,57,58,59]. The basal level of plasma GLP-2 during fasting is low, however, its concentration rises rapidly post-prandially as soon as intestinal L-cells sense carbohydrate and lipids in the lumen [60,61,62]. This quick response also implies neuronal pathway engagement. GLP-2 has a half-life of 7 minutes during which it exerts its action through binding to GLP-2R [63,64]. Depending on the GLP-2R expressing tissues (gastrointestinal tract, CNS, mesenteric fat, lymph nodes, pancreas, liver, bladder, and spleen) [65], GLP-2 employs different signaling pathways to exerts its biological effects such as production of cyclic AMP [66], increase of intracellular calcium, inhibition of glycogen synthase kinase-3, and targeted gene expression [65,67] (Figure 1A). For instance, binding of GLP-2 to its receptor in the CNS inhibits food intake [68,69] and gastric emptying through the melanocortin receptor-4 (MC4R) signaling pathway [70]. More recently, it was reported that exogenous GLP-2 inhibited postprandial gallbladder emptying in healthy men [71]. Furthermore, it was reported that GLP-2 potentiated L-type Ca2+ channel activity in primary hippocampal neurons via the activation of cAMP-dependent protein kinase A (PKA) [72] while GLP-2’s role in improving learning and memory in a mouse model of vascular dementia and a juvenile-onset diabetes in rats was mediated through ERK pathway in hippocampal neurons [73]. Interestingly, Zhang et al., demonstrated a protective role of GLP-2 analogue in a mouse model of Parkinson’s disease by suppressing the NLRP3 inflammasome and limiting mitochondrial damage [74]. In contrast to GLP-1, the role of GLP-2 in regulating lipoprotein metabolism has not been studied extensively, however, the direct action of GLP-2 is now evident. A prominent work found a stimulatory effect of GLP-2 on intestinal apoB48-containing lipoprotein secretion (apoB48) in Syrian golden hamsters which was mediated through CD36 [75]. In follow up studies, it was shown that acute administration of GLP-2 significantly elevated postprandial TG and intestinal apoB48-containing lipoproteins [61,62,75] which has been shown to be mediated through nitric oxide signaling in both mice and hamsters [62,71,75,76]. Similarly, intraperitoneal administration of GLP-2 following an intraduodenal lipid bolus resulted in elevated apoB48 levels [77]. Interestingly, a similar direct stimulatory effect of GLP-2 on postprandial CM secretion in humans has been reported [60]. In this review we will focus on recent findings related to the role of GLP-2 in the context of intestine homeostasis and inflammation and their consequences on microbiota and metabolism.

## 3. GLP-1 and GLP-2 as Regulators of Intestine Integrity and Inflammation

### 3.1. Intestinal Barrier Integrity

The primary function of the intestine is to digest and absorb nutrients and fluids, however, it represents a crossroad where the liver (through bile acids), pancreas, brain, and microbiota interact. While the gut senses nutrients and releases incretin hormones like GLP-1 and GIP [78,79,80], it also harbors the largest microbial population in the human body containing more than 100 trillion microbial cells with 3.3 million gut microbial genes that constitute the microbiome [81,82]. Structurally, the intestine functions as a multicellular barrier (enterocytes, Paneth cells, goblet cells, and enteroendocrine cells) between the external environment and the internal milieu. Therefore, maintaining a healthy intact intestine is essential for energy homeostasis and inter-organ communication.

A substantial body of evidence supports the importance of the GLP-1 system as an essential component of intestine function. GLP-1R agonism has been shown to play an important role in the protection against intestinal damage [83,84]. After 10 and 30 days of treatment, pharmacological administration of GLP-1R agonists significantly increased the weight of the small bowel and colon of normal, healthy, outbred mice; however, this effect was not associated with promoting colonic dysplasia [83]. A subsequent study found that gain and loss of GLP-1R signaling regulates mucosal expansion in the small intestine and colon [85]. These intestinotrophic actions were mediated by GLP-1R-dependent stimulation of crypt fission via Fgf7-dependent mechanisms, independent of the EGF and the intestinal epithelial IGF-1 receptors [85]. It has been revealed that GLP-1R agonists improve intestinal epithelial integrity, reduce increased gut permeability, and activate the Brunner glands, which release mucins into the intestinal lumen to create a barrier against pathogens and stomach acids [86,87,88]. Furthermore, Grunddal et al., recently found that in vivo administration of GLP-1 mimics stimulates mucin production and results in the internalization of GLP-1R in the glandular cells of the Brunner glands [6]. The distal intestine is a key location for acute L-cell response and Gcg expression which can influence the levels of GLP-1 in the blood [89]. 

In addition to GLP-1 and peptide YY (PYY), L-cells also produce ATP which activates the vagus nerve and may act synergistically with locally elevated peptide hormone concentrations [90]. Recent research on human jejunal L-cells revealed dysregulation of enteroendocrine differentiation markers associated with lower L-cell density in individuals with obesity and type 2 diabetes, which ultimately resulted in decreased GLP-1-positive cell density and may diminish the ability to produce GLP-1 in response to food [91]. According to studies from Kedees et al., and Yusta et al., Paneth cells and intestinal intraepithelial lymphocytes both express functional GLP-1Rs. Losing GLP-1R signaling worsens the effects of dextran sulphate sodium-induced intestinal damage [3,5].

GLP-2 acts a pleotropic regulator of intestine function via direct and indirect actions. Based on early data from animal models, GLP-2 was considered a promising therapeutic target for inflammatory and short bowel syndromes [59,92] because of its anti-apoptotic and proliferative activity on intestinal epithelium [56,64]. In line with these observations, GLP-2 has been shown to enhance intestinal blood flow [93] and increase surface area for absorption through increased crypt cell proliferation, intestine weight and length [94] while suppressing intestinal permeability [95,96]. Eventually, a stable analogue of GLP-2, teduglutide, has been approved to reduce parenteral support for patients with short bowel syndrome which is a life-threatening condition resulting from surgical removal of a significant mass of functional small intestine in patients with Crohn’s disease [2,97,98]. Recently, Chen et al., found a direct action of GLP-2 and its analogue on the expansion of intestinal stem cells through insulin-like growth factor signaling which further confirm the notion that GLP-2 is an intestinotrophic hormone [15].

### 3.2. Inflammation and Intestinal Failure

The mucosal immune system is complex, with inductive and effector sites based on their anatomical and functional characteristics as well as effector cells and molecules [99,100]. Effector sites consist of the lamina propria, stroma of exocrine glands, and surface epithelia [100]. These sites also contain Ag-specific mucosal effector cells, such as IgA-producing plasma cells and memory B and T cells [101], whereas inductive sites are made up of mucosa-associated lymphoid tissue and mucosa-draining lymph nodes [100] and are responsible for producing a continuous supply of memory B and T cells that are later transferred to mucosal effector sites [101].

The immune response in the gastrointestinal tract, upper respiratory, and female reproductive system is based on immune cells migrating from mucosal inductive to effector tissues via the lymphatic system [101]. Epithelial barrier integrity is maintained by mucosal immune cells, particularly intraepithelial lymphocytes (IELs) [100]. These cells support immune responses against harmful organisms and their by-products [100] by enhancing pathogen clearance and lysing pathogen-infected cells [102]. Studies have revealed that GLP-1R is functionally expressed by both Paneth cells and intestine IELs [3,5]. GLP-1R activation in immune cells reduces the production of pro-inflammatory cytokines [3,103]. Exendin-4 treatment significantly reduces pro-inflammatory cytokines IL-2, IL-17a, interferon γ, tumor necrosis factor-α at mRNA, and protein levels in IELs triggered by immobilised anti-CD3 and soluble anti-CD28 antibodies 7 through inhibiting NF-kB nuclear translocation and phosphorylation which lead to a significant suppression of proinflammatory cytokines production [104]. Through a variety of pleiotropic actions, it was found that T-cells in the gut perform vital roles in maintaining barrier integrity. In an elegant study, a subset of immune cells, subset of natural IEL immune cells (integrin β7+ natural gut intraepithelial T lymphocytes) that are scattered in the enterocyte layer of the small intestine were discovered as essential regulators of food metabolism [105]. Mice lacking natural IELs have hyperactive metabolisms and are resistant to obesity, hypercholesterolemia, hypertension, diabetes, and atherosclerosis when fed a high-fat and sugar diet. Furthermore, researchers found that IELs restrict the bioavailability of GLP-1 resulting in the endocrine mediated regulation of whole body metabolism [105] (Figure 1B).

Low-grade inflammation is a pathophysiologic factor in type 2 diabetes (T2D) and may have a significant impact on the development of diabetic complications, such as cardiovascular disease (CVD) [106,107,108]. GLP-1 suppresses inflammation and promotes gut mucosal integrity [10,109]. GLP-1 levels were elevated in mice experiencing experimental inflammation induced by the injection of cytokines or lipopolysaccharide (LPS) [110,111]. A mouse study found that administering LPS increases plasma GLP-1 levels via a Toll-like receptor 4 (TLR4)-dependent mechanism found on the basolateral membranes of enteroendocrine cells (EECs). An abrupt increase in circulating GLP-1 was caused by LPS entering EECs following dextran sodium sulphate administration to compromise the integrity of the intestinal barrier [109]. GLP-1 overexpression increased thermogenesis and polarised macrophages from the M1 to the M2 phenotype in obese mice [112]. Furthermore, GLP-1R levels elevated in critically ill patients and after inducing ischemia in the human gut [100,109,113]. According to several in vitro and in vivo studies, GLP-1 has anti-inflammatory properties in macrophages. GLP-1 was found to inhibit the secretion of IL-1β and TNF-α in cultured human monocytes as well as IL-1β and IL-6 in exendin-4-treated mice [114,115]. A later investigation revealed that GLP-1 targets inflammatory macrophages to decrease insulin resistance by blocking the NF-KB pathway and inflammatory cytokine-secreting macrophages [116]. Neutrophils and eosinophils both express GLP-1R, and GLP-IR analogue decreased markers of eosinophil activation after LPS stimulation and inhibited the production of IL-4, IL-8, and IL-13 cytokines but not IL-5 [117]. Furthermore, GLP-1R agonists prevent systemic inflammation, vascular dysfunction, and end-organ damage in mice by activating the GLP-1R in platelets through an AMP/PKA-dependent mechanism [118].

In human studies, patients with T2D have been reported to have increased inflammation and oxidative stress, which leads to insulin resistance [119,120]. Exenatide’s anti-inflammatory properties have been well studied in T2D patients [121]. Viswanathan et al., revealed that exenatide successfully manages T2D in individuals with obesity who are insulin-dependent, resulting in lowered C-reactive protein (CRP) and other metabolic parameters, including HbAlc, systolic blood pressure, and TG [122]. Furthermore, exenatide treatment for 16 weeks in T2D patients reduced levels of inflammatory markers in the blood, including high-sensitivity CRP and monocyte chemoattractant protein-1 (MCP-1), as well as the level of the oxidative stress marker 8-iso-prostaglandin F2a (PGF2a), in addition to reducing body weight and improving glucose profile and HbA1c levels [123]. Studies have shown that exenatide exerts anti-inflammatory effects independent of body weight loss in T2D patients [124,125]. Exenatide administration in T2D patients was also demonstrated to inhibit pro-inflammatory cytokines such as TNF- α, IL-1, and IL-6 in peripheral blood mononuclear cells (PBMCs) [124,125]. Furthermore, when given over a 12-week period, exenatide was found to suppress several inflammatory parameters such as ROS production by mononuclear cells (MNCs), intranuclear NFB binding, and MNC expression of TNF, JNK-1, TLR-2, TLR-4, IL-1, and SOCS-3 [124,125]. These findings could imply that GLP-1R activation can act as a beneficial immunomodulator in T2D patients.

Inflammatory bowel disease (IBD), which includes Crohn’s disease and ulcerative colitis, are multifactorial disorders characterised by immune cell infiltration and chronic inflammation-mediated relapses of the colon and the entire gastrointestinal tract, respectively [88,126]. Both Crohn’s disease and ulcerative colitis are complex disorders that are associated with the alteration of the innate and adaptive immune system, luminal and mucosa-associated microbiota, as well as epithelial function [102]. Anti-inflammatory, immunosuppressive, or biological agents are frequently used in the treatment of IBD [88,126]. However, some patients do not respond to the treatment [126]. Studies have shown that GLP-1 treatment can ameliorate dextran sulfate (DSS)-induced colitis [3,109,127]. Intraperitoneal administration of sterically stabilized phospholipid micelles coated with GLP-1 (GLP-1-SSM) for seven days in C57BL/6J mice with DSS-induced colitis resulted in decreased body weight loss and partially attenuated the diarrheal phenotype This treatment also downregulated the expression of the pro-inflammatory cytokine IL-1 β as well as prevented the depletion of the chloride anion exchanger DRA, which are both crucial for reducing diarrhea caused by IBD [127]. Moreover, Yusta et al., used a DSS colitis model in GLP-1R knockout (GLP-1R KO) mice to assess the significance of GLP-1 signaling in a localized inflammatory context. The authors revealed that lack of GLP-1R signaling worsens the effect of DSS-induced intestinal damage [3]. The GLP-1R KO mice exhibited much worse colon damage, significantly higher disease activity scores, and much more weight loss in response to DSS-induced colitis than wild-type controls. Additionally, trefoil factor-3 and interferon-gamma gene expression in GLP-1R KO mice + DSS were dysregulated but transforming growth factor beta-2 expression was elevated [3].

Furthermore, in severe combined immunodeficient mice (SCID) mice injected with BALB/C CD4+ T cells, treatment with the GLP-1 agonist liraglutide alleviated colitis by significantly improving colon weight to length ratios, reducing histopathological score, and lowering pro-inflammatory cytokine levels such as CCL20, IL-33, and IL-22 [86]. Studies have shown that patients with ulcerative colitis who underwent ileostomy or colectomy have reduced postprandial GLP-1 response [128,129]. Subsequent research revealed that GLP-1R mRNA was reduced in samples obtained from inflamed areas of the colon in IBD patients [86]. In contrast, compared to controls, GLP-1 was increased in the serum of IBD patients [130]. In a case report of a patient with ulcerative colitis receiving GLP-1 treatment, daily subcutaneous liraglutide injections led to a full remission of colitis symptoms [128].

In addition to its protective and growth promoting roles in the intestine, GLP-2 exerts anti-inflammatory effects through multiple different mechanisms. For instance, GLP-2 dampened pro-inflammatory cytokine production in LPS-treated macrophages through inhibiting NF-κB activity and ERK phosphorylation [131] and reducing mucosal inflammatory responses [132] in mice, while in a model of obstructive jaundice in rats, GLP-2 reduced serum levels of TGF-β1, bilirubin, and endotoxin, which improved intestinal barrier function [133]. Interestingly, data from animal models of intestinal barrier dysfunction in mice, rats, and piglets underlines the importance and protective action of GLP-2 in inflammation-mediated intestine injuries [58,134,135]. More recently, it was found that patients who develop acute graft-versus-host disease (GVHD) also have dysbiosis and lower levels of GLP-2. This association was further validated using teduglutide, which upon administration showed a beneficial outcome by reducing de novo acute GVHD and steroid refractory GVHD in multiple mouse models. The underlying mechanism could be attributed to GLP-2’s anti-apoptotic action along with its regenerative impact on Paneth cells and intestine stem cells [136]. While parenteral nutrition remains the life-saving standard for nutrient delivery in patients with intestinal failure, serious complications may develop such as liver and intestinal mucosal barrier injury, inflammation, and bacterial translocation [137,138]. To further understand the role of GLP-2 to ameliorate parenteral nutrition-associated gut injury, using a mouse model of parenteral nutrition, Deng et al., showed that GLP-2 significantly suppressed the expression of pro-inflammatory cytokines TNF-a and IL-6 in the ileum and reduced serum levels of D-lactic acids and LPS [132].

The majority of IBD research has been conducted using animal models, and there are few clinical investigations. Considering that GLP-1 and GLP-2 are crucial for intestinal healing after injury and may have potential anti-inflammatory and metabolic effects. Therefore, it appears that the production and regulation of GLPs will facilitate in the treatment of IBD.

## 4. GLP-1 and GLP-2 Mediate Gut Microbiota–Immune System Crosstalk

### 4.1. Gut Microbiota and Immune System Interaction

The interaction between the immune system and microbiota has been extensively reviewed elsewhere [21,139,140,141], and for the purpose of this review, the focus will be on the roles of GLP-1 and GLP-2 in orchestrating such crosstalk in the intestine. Microbiota and their metabolites come in direct contact with intestine through mucosal surfaces where food is further processed and absorbed [20]. The structural components of the intestinal lining not only support innate defence against pathogenic bacteria and inflammatory mediators, but they also allow commensal intestinal microbiota to coexist through a complex and dynamic communication between gut receptors, immune cells, and nerve cells [100,142,143]. It is widely known that the microbiota closely relates to human health and the development of diseases.

The intestinal microbiota, which consists of billions of microorganisms, lives in the gastrointestinal tract where endogenous GLP-1 is produced [109]. The host and gut microbiota interaction is mediated through the detection of microbial-related molecular patterns, which frequently comprise bacterial antigens such as unmethylated bacterial DNA, LPS, capsular polysaccharides, muramic acid, and flagellin [144]. 

Some of these bacteria produce pro-inflammatory LPS, which affects hormone release and gut immunity [109]. It has been suggested that EECs play an important role in directing immune responses against pathogens and commensal microorganisms [145]. Interactions between EECs and bacteria aid in the maintenance of intestinal immune homeostasis because EECs have an innate immune signalling pathway and express GPCRs that allow them to recognise microbe-associated products [146]. In response to exposure to molecular patterns associated with microbes (MAMPS), mammalian EECs express TLRs and initiate an NF-κB-mediated response [146]. Moreover, EECs can detect the presence of LPS or flagellin in the lumen. These cells can also produce CXCL1, CXCL3, and CCL20, which are chemoattractant molecules that can draw immune cells from the lamina propria and activate them through NK4/IL-32 stimulation, causing them to produce TNF- α via NOD2 and aggravate the inflammatory state [147].

The gut microbiota has been shown to function as an endocrine organ, producing and regulating numerous compounds that reach the circulation and act to influence the function of distal organs and systems, actively taking part in amino acid generation, pathogen displacement [148], as well as circadian rhythmicity, nutritional responses, regulating metabolic activities and immune function [21,149,150]. The gut microbiota is crucial in the regulation of many different aspects of metabolic disease, especially through the production of a myriad of metabolites and their interactions with receptors on host cells, which can activate or inhibit signalling pathways [20,27]. The microbial composition impacts the abundance and availability of metabolites [19,151]. Additionally, the composition of microbiota is highly flexible in response to diet and can undergo rapid and significant change [148]. Accumulating studies suggest that high-fat and high-fructose diet can alter the Firmicutes /Bacteroidetes ratio, which are dominant bacterial gut phyla with a causal link to obesity and T2D phenotypes [152,153].

The colonization of the gut microbiota begins at birth and continues to evolve, resulting in a highly complex microbiota profile with thousands of functional taxonomic units [154]. An initial study showed that the lack of commensal microorganisms in germ-free (GF) mice causes intense intestine abnormalities in the lymphoid tissue architecture and immune system [155]. Previously, abnormally high serum IgE levels in GF mice were reported [154,156], and these elevated IgE levels increased mast-cell surface-bound IgE and heightened systemic anaphylaxis [154]. Therefore, intestinal microbial diversity during early-life colonization is essential to build an immune-regulatory network that protects against the generation of mucosal IgE, which is connected to allergy susceptibility [154]. IgA antibodies and IELs (αβ and γδ) were both considerably decreased in GF mice and substantially stimulated following colonization [157,158]. Short chain fatty acids (SCFA) levels are known to be low in GF animals [159]. Additionally, GF mice lack the potent immunomodulatory effector cells known as IL-17+CD4+ T (Th17) cells, which are abundant in the lamina propria of the small intestine and recover after microbial colonization [21,160]. Furthermore, a study demonstrated that increased morbidity in IBD and allergic asthma models occurs in GF mice as compared to specific pathogen-free mice (SPF) because invariant natural killer T (iNKT) cells accumulate in the intestinal lamina propria and lung; this phenomenon was also associated elevated intestinal and pulmonary production of the chemokine ligand CXCL16 [161].

Gut-associated lymphoid tissues (GALTs) are part of mucosa-associated lymphoid tissues (MALTs), which recognize pathogens, initiate innate immune responses, and present antigens to stimulate the adaptive immune system. It was discovered that the gut microbiota is crucial for the structural development of GALTs as the GALT development is abnormal in GF mice, as evidenced by the aberrant formation of crypt patches and isolated lymphoid follicles (ILFs) [162]. The gut microbiota influences the structural development of GALT and Peyer’s patches through epigenetic modulators such as short chain fatty acids (SCFAs) as well as TLR pathways that promote the production of antimicrobial peptides such as REGIIIβ and REGIIIγ [162]. Recent research investigated the interactions between the macrophages and the commensal microbiota. A large polysaccharide derived from the microbiota has been shown to induce an anti-inflammatory gene signature in murine intestinal macrophages [163].

The intestinal epithelium is protected from local bacteria by a thick coating of mucus and tight junction, which are key components in limiting trans-epithelial permeability. Tight junctions and related cytoskeletal proteins are upregulated in response to microbial signals such as those delivered by the metabolite indole, which in turn helps to strengthen the epithelial barrier [164]. Paneth cells, which are specialized secretory cells of the small intestinal mucosa, are responsible for producing most intestinal antimicrobial peptides that interact with the microbiota [165,166]. Paneth cells produce α-defensins that regulate microbiota composition and are essential for intestinal barrier function and homeostasis [166]. Additionally, mucosal barrier function is maintained by secretory IgA antibodies and antimicrobial peptides (AMPs) [167,168]. It has been demonstrated that, in addition to IgA antibodies, IgG antibodies can react to microbiota. In response to commensal and enteropathogenic microbes, intestinal IgA+ and IgG+ plasmablasts express antigen-specific antibodies [169].

The direct communication between microbiota and L-cells is mediated through functional TLRs, which play a role in pathogen recognition as well as the initiation of inflammatory and immune responses [159,170]. The interaction of commensal bacterial products with host microbial pattern recognition receptors (PRRs), such as TLRs, is critical for epithelial integrity and pathogen defence [171]. TLRs can be activated by microbial compounds, which then triggers signalling pathways that cause the production of antimicrobial genes and inflammatory cytokines [170]. TLR5 and nucleotide-binding oligomerization domain-containing protein 1 (NOD1) have been suggested to shape the composition of the gut microbiota [167,172,173]. Furthermore, NOD1 aids in the development of adaptive lymphoid tissues as well as the maintenance of intestinal homeostasis [167,172,173]. 

The main metabolites produced in the colon by bacterial fermentation of dietary fibers are SCFAs such as acetate, propionate, and butyrate [28]. Dietary fiber rich diets are linked to reduced inflammation due to increased SCFA synthesis and activation of GPCR [24]. SCFAs activate signalling pathways via GPCRs: GPR41, GPR43, and GPR109A [24]. These GPCRs are crucial for preventing NF-kB activation in IECs and immune cells, and SCFAs have also been shown to inhibit NF-kB and lead to decreased inflammatory cytokine production [24,29]. SCFAs suppress IL-8 secretion and expression [25] and butyrate has also been shown to induce colonic Treg [174]. Nonobese diabetic (NOD) mouse models customised diets leading to enhanced bacterial production of acetate and butyrate, resulted in nearly complete protection from T1D, mostly because of the immune-modulating properties of SCFAs [29,174,175,176,177]. Butyrate can also boost host defence against pathogens by inhibiting the enzyme histone deacetylase 3 (HDAC3), which promotes monocyte-to-macrophage differentiation [176].

Furthermore, tryptophane catabolites has been shown to influence immune responses by binding the aryl hydrocarbon receptor (AhR), which is abundant at mucosal surfaces. When AhR is activated, it improves intestinal epithelial barrier function as well as regulates inflammation by lowering epithelial stress and gut immunity [24]. Trimethylamine N-oxide (TMAO), a soluble microbiome-derived metabolite, has recently been shown to promote murine macrophage polarisation via the NLRP3 inflammasome [178] (Figure 1B). 

Dysbiosis of gut microbiota has been linked to a variety of changes in the immune system [21,162]. Microbiota dysbiosis has also been linked to IBD through its impact on inflammation and the intestinal barrier [23]. It was reported that the composition of gut microbiota differs in IBD patients compared to healthy subjects as the ratio of Bacteroidetes to Firmicutes decreases while the abundance of gammaproteobacterial increases [26,179]. Furthermore, IBD is associated with decreased abundance of Bacteroides, Eubacterium, and Lactobacillus [180]. The butyrate producer Faecalibacterium prausnitzii has been linked to maintaining gut mucosal health. Butyrate improves IBD by being the primary source of energy for colonocytes, improving epithelial barrier integrity, and inhibiting inflammation [181].

### 4.2. GLP-1/GLP-2 and Microbiota Crosstalk: Interdependent Relationship

It has been demonstrated that the microbiota stimulates EECs, prompting them to secrete hormones and control gastrointestinal tract motility [182]. In vitro studies on colonic cultures [183,184,185] or using isolated perfused rat colon [186] showed that microbiota-derived metabolites such as acetate, propionate, and butyrate enhanced GLP-1 secretion. Interestingly, indole, a bacterial metabolite, showed a biphasic action on GLP-1 secretion. While short-term effect of indole enhanced GLP-1 release, the prolonged exposure suppressed its secretion which imply an adaptive role for GLP-1 to modulate host metabolism [187]. Further support to this hypothesis has been reported in GF mice models. Antibiotic-treated animals and GF mice were shown to have higher plasma GLP-1 levels than controls [188] to adapt intestinal transit in response to energy inadequacy in colon. Furthermore, prebiotics have recently been shown to raise GLP-1 and PYY and decrease ghrelin release in humans [182].

In a clinical study, rectal and intravenous infusion of acetate elevated GLP-1 and PYY levels in overweight and hyperinsulinemic women [188]. Colonic L cells express FFA receptors 2 and 3 (FFAR2 and FFAR3) that are involved in SCFA-stimulated GLP-1 production by increasing intracellular calcium through G_q_- or G_i/o_ and G_i_ signaling [187]. Studies have shown that FFAR2 & FFAR3 KO mice exhibit considerably lower basal levels of GLP-1 or SCFA-induced GLP-1 release than wild-type control mice. However, it was postulated that FFAR3 may be less responsible for the release of GLP-1 compared to FFAR2 because FFAR3 agonism had no effect on secretion of GLP-1 [182,189]. 

GLP-1R agonists have been demonstrated in preclinical and clinical trials to mediate alterations in the gut microbiota [190,191,192,193,194,195]. GLP-1R agonists were reported to affect the composition of the microbiota through altering the rate and time of gastric emptying, as well as the internal environment of the gut lumen, including local pH levels and nutrient availability [196]. Liraglutide administration was shown in mouse and rat studies to significantly reduce the relative abundance of microbial phenotypes associated with obesity while enhancing phenotypes associated with leanness [190,197]. In obese and T2D/obese rat models, liraglutide administration significantly increased the Bacteroidetes/Firmicutes ratio as well as reduced the abundance of obesity-related phylotypes such as *Romboutsia, Ruminiclostridium, and Erysipelotrichaceae* while increasing lean-related phylotypes like *Prevotella* [190]. 

A study showed that liraglutide, but not saxagliptin, alters the gut microbiome in mice. Liraglutide reduced obesity-related phylotypes in mice regardless of whether they were correlated negatively or positively [190,197].

Liraglutide modifies the gut microbiota, increasing the abundance of the genera *Allobaculum*, *Turicibacter*, *Anaerostipes, Blautia*, *Lactobacillus*, *Butyricimonas*, and *Desulfovibrio* while decreasing the abundance of the order Clostridiales (phylum Firmicutes) and Bacteroidales (phylum Bacteroidetes) (209). However, no discernible changes in the abundance of the phyla Firmicutes and Bacteroides were observed [190,197]. A subsequent study showed that administration of liraglutide and dual GLP-1R/GLP-2R agonist GUB09-145 in diet-induced obese mice decreased the abundance of Firmicutes (*Lachnospiraceae*, *Clostridiales*) and increased the abundance of Proteobacteria (e.g., *Burkholderiales bacterium YL45*) and Verrucomicrobia (e.g., *Akkermansia muciniphila*) as well as Firmicutes (*Clostridiales*, *Oscillospiraceae*) [191]. The effects of liraglutide and GUB09-145 were primarily linked to improved bacterial lipid handling and sulfur metabolism. These results suggested that GLP-1R agonists can prevent weight gain by modulating the gut microbiota composition.

The effect of GLP-1R agonists on gut microbiota has been studied in clinical trials in T2D patients. Increased levels of deoxycholic acid, a secondary bile acid produced by bacterial metabolism, were observed in T2D patients treated with liraglutide but not sitagliptin or a comparable placebo, suggesting changes in the gut flora [198]. Additionally, patients who had diabetes for a long time had a significant reduction in *Akkermansia*, whereas subjects given a GLP-1 agonist had more *Akkermansia* than those given metformin [194]. *A. muciniphila* has been shown to improve the integrity of gut barrier function in rodents [199]. Importantly, GLP-1 level was positively correlated with *Akkermansia* sp. abundance in patients with obesity after gastric bypass surgery and dietary intervention [200,201]. A recent study found a significant difference in the Beta diversity of the gut microbiota between GLP-1 R agonist responder and non-responder patients with T2D [202]. Decreased levels of HbA1c in the responder group correlated with increase abundance of *Bacteroides dorei*, *Lachnoclostridium* sp., *Mitsuokella multacida*, and *Prevotella copri* ^202^. The representative positive signatures for GLP-1R agonist hyperglycemia responses were *Bacteroides dorei* and *Lachnoclostridium* sp., whereas the representative negative signature was *Mitsuokella multacida*. They also observed that *Bacteroides dorei* and *Roseburia inulinivorans* were more abundant, indicating that GLP-1R agonist responders have reduced inflammation because of their role in enhancing gut barrier function and production of butyrate-derived metabolites [202]. Over last few years, scientific breakthroughs have expanded our understanding on an interesting communication circuit where microbiota, intestine, liver, and brain are interconnected to regulate metabolism and energy expenditure [203,204,205,206]. In this context, although the data are limited, they point to an interdependent relationship between microbiota and GLP-2 in metabolic disease. For instance, prebiotic (oligofructose) treatment in mice enhanced GLP-2 secretion and reversed high-fat diet-associated alterations like increased gut permeability as well as high levels of circulating endotoxins and pro-inflammatory cytokines. Interestingly, these effects were GLP-2 dependent [207]. Another study in diet-induced obese rats showed that probiotic *Bifidobacterium animalis* elevated GLP-2 levels [95]. Moreover, in patients with ulcerative colitis, serum level of GLP-2 was associated with microbiota diversity and abundance [208]. The direct impact of GLP-2 on shaping microbiota in young and old rats has now been reported to be essential for intestine integrity and energy homeostasis [209]. Importantly, similar effects have been reported in glucose-tolerant subjects where the beneficial effects of probiotic *Lactobacillus reuteri* ingestion for four weeks was mediated through an increase in GLP-1 and GLP-2 secretion [210].

## 5. Conclusions

The discovery and translational implications of GLP-1 and -2 significantly impacted the clinical outcomes, especially in treating patients with type 2 diabetes. The beneficial effects of GLP-1 have been attributed to its roles in improving intestinal barrier functions through stimulating crypt cell fission, downregulating proinflammatory cytokines by immune cells, and modulating gut microbiota. However, GLP-2 and its agonists (e.g., teduglutide) employ different signaling mechanisms to protect the intestine from injury. For instance, GLP-2 expands intestinal stem cells numerically and promotes intestinal epithelial proliferation. Furthermore, it inhibits NLPR3 inflammasome activation, enhances the tight junction, and limits the release of LPS into the circulation. GLP-1 and -2 act as sensors for metabolic and inflammatory cues and play orchestrating roles in maintaining gut health and metabolism through a complex network of reciprocal communications with microbiota and the immune system. Therefore, based on the growing understanding of the roles of GLP-1 and -2 and the expression of their receptors on different tissues, uncharacterized additional roles of these hormones may be revealed in the near future.

## Data Availability

Not applicable.

## References

[1] Baggio L.L., Drucker D.J. (2007). Biology of incretins: GLP-1 and GIP. Gastroenterology.

[2] Drucker D.J., Habener J.F., Holst J.J. (2017). Discovery, characterization, and clinical development of the glucagon-like peptides. J. Clin. Investig..

[3] Yusta B., Baggio L.L., Koehler J., Holland D., Cao X., Pinnell L.J., Johnson-Henry K.C., Yeung W., Surette M.G., Bang K.W.A. (2015). GLP-1R agonists modulate enteric immune responses through the intestinal intraepithelial lymphocyte GLP-1R. Diabetes.

[4] Pyke C., Heller R.S., Kirk R.K., Ørskov C., Reedtz-Runge S., Kaastrup P., Hvelplund A., Bardram L., Calatayud D., Knudsen L.B. (2014). GLP-1 receptor localization in monkey and human tissue: Novel distribution revealed with extensively validated monoclonal antibody. Endocrinology.

[5] Kedees M.H., Guz Y., Grigoryan M., Teitelman G. (2013). Functional activity of murine intestinal mucosal cells is regulated by the glucagon-like peptide-1 receptor. Peptides.

[6] Grunddal K.V., Jensen E.P., Ørskov C., Andersen D.B., Windeløv J.A., Poulsen S.S., Rosenkilde M.M., Knudsen L.B., Pyke C., Holst J.J. (2022). Expression Profile of the GLP-1 Receptor in the Gastrointestinal Tract and Pancreas in Adult Female Mice. Endocrinology.

[7] Egan J.M., Bulotta A., Hui H., Perfetti R. (2003). GLP-1 receptor agonists are growth and differentiation factors for pancreatic islet beta cells. Diabetes/Metab. Res. Rev..

[8] Talsania T., Anini Y., Siu S., Drucker D.J., Brubaker P.L. (2005). Peripheral exendin-4 and peptide YY^3–36^ synergistically reduce food intake through different mechanisms in mice. Endocrinology.

[9] Kahles F., Meyer C., Möllmann J., Diebold S., Findeisen H.M., Lebherz C., Trautwein C., Koch A., Tacke F., Marx N. (2014). GLP-1 secretion is increased by inflammatory stimuli in an IL-6–dependent manner, leading to hyperinsulinemia and blood glucose lowering. Diabetes.

[10] Ørgaard A., Holst J.J. (2017). The role of somatostatin in GLP-1-induced inhibition of glucagon secretion in mice. Diabetologia.

[11] Horne R.G., Yu Y., Zhang R., Abdalqadir N., Rossi L., Surette M., Sherman P.M., Adeli K. (2020). High fat-high fructose diet-induced changes in the gut microbiota associated with dyslipidemia in Syrian hamsters. Nutrients.

[12] Tsai C.H., Hill M., Asa S.L., Brubaker P.L., Drucker D.J. (1997). Intestinal growth-promoting properties of glucagon-like peptide-2 in mice. Am. J. Physiol. Endocrinol. Metab..

[13] Thulesen J., Hartmann B., Hare K.J., Kissow H., Ørskov C., Holst J.J., Poulsen S.S. (2004). Glucagon-like peptide 2 (GLP-2) accelerates the growth of colonic neoplasms in mice. Gut.

[14] Estall J.L., Drucker D.J. (2006). Glucagon-like peptide-2. Annu. Rev. Nutr..

[15] Brubaker P.L. (2011). Glucagon-like peptide-2 and the regulation of intestinal growth and function. Compr. Physiol..

[16] Chen M.E., Naeini S.M., Srikrishnaraj A., Drucker D.J., Fesler Z., Brubaker P.L. (2022). Glucagon-like peptide-2 stimulates S-phase entry of intestinal Lgr5 + stem cells. Cell. Mol. Gastroenterol. Hepatol..

[17] Thursby E., Juge N. (2017). Introduction to the human gut microbiota. Biochem. J..

[18] Durack J., Lynch S.V. (2019). The gut microbiome: Relationships with disease and opportunities for therapy. J. Exp. Med..

[19] Cani P.D., Moens de Hase E., Van Hul M. (2021). Gut microbiota and host metabolism: From proof of concept to therapeutic intervention. Microorganisms.

[20] de Vos W.M., Tilg H., Van Hul M., Cani P.D. (2022). Gut microbiome and health: Mechanistic insights. Gut.

[21] Zheng D., Liwinski T., Elinav E. (2020). Interaction between microbiota and immunity in health and disease. Cell Res..

[22] Wang B., Yao M., Lv L., Ling Z., Li L. (2017). The human microbiota in health and disease. Engineering.

[23] Kaijian H., Zhuo-Xun W., Xuan-Yu C., Jing-Quan W., Dongya Z., Chuanxing X., Zhu D., Koya J.B., Liuya W., Li J. (2022). Microbiota in health and diseases. Signal Transduct. Target. Ther..

[24] Gasaly N., De Vos P., Hermoso M.A. (2021). Impact of bacterial metabolites on gut barrier function and host immunity: A focus on bacterial metabolism and its relevance for intestinal inflammation. Front. Immunol..

[25] Asarat M., Vasiljevic T., Apostolopoulos V., Donkor O. (2015). Short-chain fatty acids regulate secretion of IL-8 from human intestinal epithelial cell lines in vitro. Immunol. Investig..

[26] Morgan X.C., Tickle T.L., Sokol H., Gevers D., Devaney K.L., Ward D.V., Reyes J.A., Shah S.A., LeLeiko N., Snapper S.B. (2012). Dysfunction of the intestinal microbiome in inflammatory bowel disease and treatment. Genome Biol..

[27] Krautkramer K.A., Fan J., Bäckhed F. (2021). Gut microbial metabolites as multi-kingdom intermediates. Nat. Rev. Microbiol..

[28] Pascale A., Marchesi N., Marelli C., Coppola A., Luzi L., Govoni S., Giustina A., Gazzaruso C. (2018). Microbiota and metabolic diseases. Endocrine.

[29] Usami M., Kishimoto K., Ohata A., Miyoshi M., Aoyama M., Fueda Y., Kotani J. (2008). Butyrate and trichostatin A attenuate nuclear factor κB activation and tumor necrosis factor Î± secretion and increase prostaglandin E2 secretion in human peripheral blood mononuclear cells. Nutr. Res..

[30] Azmy Nabeh O., Ishak Attallah M., El-Sayed El-Gawhary N. (2020). The pivotal relation between glucagon-like peptides, NFκB and inflammatory bowel disease. Clin. Exp. Pharmacol. Physiol..

[31] Marathe C.S., Rayner C.K., Jones K.L., Horowitz M. (2013). Glucagon-like peptides 1 and 2 in health and disease: A review. Peptides.

[32] Rouillé Y., Martin S., Steiner D.F. (1995). Differential processing of proglucagon by the subtilisin-like prohormone convertases PC2 and PC3 to generate either glucagon or glucagon-like peptide. J. Biol. Chem..

[33] Furuta M., Yano H., Zhou A., Rouillé Y., Holst J.J., Carroll R., Ravazzola M., Orci L., Furuta H., Steiner D.F. (1997). Defective prohormone processing and altered pancreatic islet morphology in mice lacking active SPC2. Proc. Natl. Acad. Sci. USA.

[34] Damholt A.B., Buchan A.M., Holst J.J., Kofod H. (1999). Proglucagon processing profile in canine L cells expressing endogenous prohormone convertase 1/3 and prohormone convertase 2. Endocrinology.

[35] Lafferty R.A., O’Harte F.P.M., Irwin N., Gault V.A., Flatt P.R. (2021). Proglucagon-Derived Peptides as Therapeutics. Front. Endocrinol..

[36] Lindquist P., Madsen J.S., Bräuner-Osborne H., Rosenkilde M.M., Hauser A.S. (2021). Mutational Landscape of the Proglucagon-Derived Peptides. Front. Endocrinol..

[37] Holst J.J. (2022). Glucagon and other proglucagon-derived peptides in the pathogenesis of obesity. Front. Nutr..

[38] Jacobson A., Yang D., Vella M., Chiu I.M. (2021). The intestinal neuro-immune axis: Crosstalk between neurons, immune cells, and microbes. Mucosal. Immunol..

[39] Cho Y.M., Merchant C.E., Kieffer T.J. (2012). Targeting the glucagon receptor family for diabetes and obesity therapy. Pharmacol. Ther..

[40] Drucker D.J., Nauck M.A. (2006). The incretin system: Glucagon-like peptide-1 receptor agonists and dipeptidyl peptidase-4 inhibitors in type 2 diabetes. Lancet.

[41] Meier J.J. (2012). GLP-1 receptor agonists for individualized treatment of type 2 diabetes mellitus. Nat. Rev. Endocrinol..

[42] Xu G., Stoffers D.A., Habener J.F., Bonner-Weir S. (1999). Exendin-4 stimulates both beta-cell replication and neogenesis, resulting in increased beta-cell mass and improved glucose tolerance in diabetic rats. Diabetes.

[43] Hellström P.M. (2009). GLP-1: Broadening the incretin concept to involve gut motility. Regul. Pept..

[44] Abbott C.R., Monteiro M., Small C.J., Sajedi A., Smith K.L., Parkinson J.R.C., Ghatei M.A., Bloom S.R. (2005). The inhibitory effects of peripheral administration of peptide YY^3-36^ and glucagon-like peptide-1 on food intake are attenuated by ablation of the vagal-brainstem-hypothalamic pathway. Brain Res..

[45] Pi-Sunyer X., Astrup A., Fujioka K., Greenway F., Halpern A., Krempf M., Lau D.C.W., Le Roux C.W., Violante Ortiz R., Jensen C.B. (2015). A randomized, controlled trial of 3.0 mg of liraglutide in weight management. N. Engl. J. Med..

[46] Kelly A.S., Auerbach P., Barrientos-Perez M., Gies I., Hale P.M., Marcus C., Mastrandrea L.D., Prabhu N., Arslanian S. (2020). A randomized, controlled trial of liraglutide for adolescents with obesity. N. Engl. J. Med..

[47] Hsieh J., Longuet C., Baker C.L., Qin B., Federico L.M., Drucker D.J., Adeli K. (2010). The glucagon-like peptide 1 receptor is essential for postprandial lipoprotein synthesis and secretion in hamsters and mice. Diabetologia.

[48] Xiao C., Dash S., Morgantini C., Patterson B.W., Lewis G.F. (2014). Sitagliptin, a DPP-4 inhibitor, acutely inhibits intestinal lipoprotein particle secretion in healthy humans. Diabetes.

[49] Tremblay A.J., Lamarche B., Deacon C.F., Weisnagel S.J., Couture P. (2011). Effect of sitagliptin therapy on postprandial lipoprotein levels in patients with type 2 diabetes. Diabetes Obes. Metab..

[50] Xiao C., Bandsma R.H.J., Dash S., Szeto L., Lewis G.F. (2012). Exenatide, a glucagon-like peptide-1 receptor agonist, acutely inhibits intestinal lipoprotein production in healthy humans. Arterioscler. Thromb. Vasc. Biol..

[51] Matikainen N., MÃ¤nttÃ¤ri S., Schweizer A., Ulvestad A., Mills D., Dunning B.E., Foley J.E., Taskinen M.R. (2006). Vildagliptin therapy reduces postprandial intestinal triglyceride-rich lipoprotein particles in patients with type 2 diabetes. Diabetologia.

[52] Farr S., Baker C., Naples M., Taher J., Iqbal J., Hussain M., Adeli K. (2015). Central nervous system regulation of intestinal lipoprotein metabolism by glucagon-like peptide-1 via a brain-gut axis. Arterioscler. Thromb. Vasc. Biol..

[53] Varin E.M., Hanson A.A., Beaudry J.L., Nguyen M.-A., Cao X., Baggio L.L., Mulvihill E.E., Drucker D.J. (2020). Hematopoietic cell–versus enterocyte-derived dipeptidyl peptidase—4 differentially regulates triglyceride excursion in mice. JCI Insight.

[54] Richards P., Parker H.E., Adriaenssens A.E., Hodgson J.M., Cork S.C., Trapp S., Gribble F.M., Reimann F. (2014). Identification and characterization of GLP-1 receptor–expressing cells using a new transgenic mouse model. Diabetes.

[55] Nahmias A., Stahel P., Tian L., Xiao C., Lewis G.F. (2021). GLP-1 (glucagon-like peptide-1) is physiologically relevant for chylomicron secretion beyond its known pharmacological role. Arterioscler. Thromb. Vasc. Biol..

[56] Drucker D.J., Erlich P., Asa S.L., Brubaker P.L. (1996). Induction of intestinal epithelial proliferation by glucagon-like peptide 2. Proc. Natl. Acad. Sci. USA.

[57] Buchman A.L., Katz S., Fang J.C., Bernstein C.N., Abou-Assi S.G., Teduglutide Study G. (2010). Teduglutide, a novel mucosally active analog of glucagon-like peptide-2 (GLP-2) for the treatment of moderate to severe Crohn’s disease. Inflamm. Bowel Dis..

[58] Reiner J., Thiery J., Held J., Berlin P., Skarbaliene J., Vollmar B., Jaster R., Eriksson P.O., Lamprecht G., Witte M. (2022). The dual GLP-1 and GLP-2 receptor agonist dapiglutide promotes barrier function in murine short bowel. Ann. N.Y. Acad. Sci..

[59] Eliasson J., Hvistendahl M.K., Freund N., Bolognani F., Meyer C., Jeppesen P.B. (2022). Apraglutide, a novel glucagon-like peptide-2 analog, improves fluid absorption in patients with short bowel syndrome intestinal failure: Findings from a placebo-controlled, randomized phase 2 trial. J. Parenter. Enter. Nutr..

[60] Dash S., Xiao C., Morgantini C., Connelly P.W., Patterson B.W., Lewis G.F. (2014). Glucagon-like peptide-2 regulates release of chylomicrons from the intestine. Gastroenterology.

[61] Hein G.J., Baker C., Hsieh J., Farr S., Adeli K. (2013). GLP-1 and GLP-2 as yin and yang of intestinal lipoprotein production: Evidence for predominance of GLP-2-stimulated postprandial lipemia in normal and insulin-resistant states. Diabetes.

[62] Hsieh J., Trajcevski K.E., Farr S.L., Baker C.L., Lake E.J., Taher J., Iqbal J., Hussain M.M., Adeli K. (2015). Glucagon-Like Peptide 2 (GLP-2) Stimulates Postprandial Chylomicron Production and Postabsorptive Release of Intestinal Triglyceride Storage Pools via Induction of Nitric Oxide Signaling in Male Hamsters and Mice. Endocrinology.

[63] Hartmann B., Harr M.B., Jeppesen P.B., Wojdemann M., Deacon C.F., Mortensen P.B., Holst J.J. (2000). In vivo and in vitro degradation of glucagon-like peptide-2 in humans. J. Clin. Endocrinol. Metab..

[64] Drucker D.J. (2001). Glucagon-like peptide 2. J. Clin. Endocrinol. Metab..

[65] Yusta B., Matthews D., Koehler J.A., Pujadas G., Kaur K.D., Drucker D.J. (2019). Localization of glucagon-like peptide-2 receptor expression in the mouse. Endocrinology.

[66] Velázquez E., Blázquez E., Ruiz-Albusac J.M. (2012). Glucagon-like peptide-2 (GLP-2) modulates the cGMP Signalling pathway by regulating the expression of the soluble guanylyl cyclase receptor subunits in cultured rat astrocytes. Mol. Neurobiol..

[67] Munroe D.G., Gupta A.K., Kooshesh F., Vyas T.B., Rizkalla G., Wang H., Demchyshyn L., Yang Z.-J., Kamboj R.K., Chen H. (1999). Prototypic G protein-coupled receptor for the intestinotrophic factor glucagon-like peptide 2. Proc. Natl. Acad. Sci. USA.

[68] Sun W., Chen L.-N., Zhou Q., Zhao L.-H., Yang D., Zhang H., Cong Z., Shen D.-D., Zhao F., Zhou F. (2019). A unique hormonal recognition feature of the human glucagon-like peptide-2 receptor. Cell Res..

[69] Sun L., Pang Y., Wang X., Wu Q., Liu H., Liu B., Liu G., Ye M., Kong W., Jiang C. (2019). Ablation of gut microbiota alleviates obesity-induced hepatic steatosis and glucose intolerance by modulating bile acid metabolism in hamsters. Acta Pharm. Sin. B.

[70] Sun H., Meng K., Hou L., Shang L., Yan J. (2021). GLP-2 decreases food intake in the dorsomedial hypothalamic nucleus (DMH) through Exendin (9–39) in male Sprague-Dawley (SD) rats. Physiol. Behav..

[71] Hansen N.L., Brønden A., Nexøe-Larsen C.C., Christensen A.S., Sonne D.P., Rehfeld J.F., Wever Albretchsen N.J., Hartmann B., Vilsbøll T., Holst J.J. (2020). Glucagon-like peptide 2 inhibits postprandial gallbladder emptying in man: A randomized, double-blinded, crossover study. Clin. Transl. Gastroenterol..

[72] Wang Y., Guan X. (2010). GLP-2 potentiates L-type Ca^2+^ channel activity associated with stimulated glucose uptake in hippocampal neurons. Am. J. Physiol. Endocrinol. Metab..

[73] Sasaki-Hamada S., Fujiwara A., Iwai T., Oka J.-I. (2021). GLP-2 restores impairments in spatial working memory and hippocampal LTD via the MEK/ERK pathway in juvenile-onset diabetes rats. Behav. Brain Res..

[74] Zhang Z., Hao L., Shi M., Yu Z., Shao S., Yuan Y., Zhang Z., Hölscher C. (2021). Neuroprotective Effects of a GLP-2 Analogue in the MPTP Parkinson’s Disease Mouse Model. J. Park. Dis..

[75] Hsieh J., Longuet C., Maida A., Bahrami J., Xu E., Baker C.L., Brubaker P.L., Drucker D.J., Adeli K. (2009). Glucagon-like peptide-2 increases intestinal lipid absorption and chylomicron production via CD36. Gastroenterology.

[76] Grande E.M., Raka F., Hoffman S., Adeli K. (2022). GLP-2 Regulation of Dietary Fat Absorption and Intestinal Chylomicron Production via Neuronal Nitric Oxide Synthase (nNOS) Signaling. Diabetes.

[77] Stahel P., Xiao C., Davis X., Tso P., Lewis G.F. (2019). Glucose and GLP-2 (Glucagon-Like Peptide-2) mobilize intestinal triglyceride by distinct mechanisms. Arterioscler. Thromb. Vasc. Biol..

[78] Greenwood-Van Meerveld B., Johnson A.C., Grundy D. (2017). Gastrointestinal Physiology and Function. Handb Exp Pharm..

[79] Wang S.Z., Yu Y.J., Adeli K. (2020). Role of Gut Microbiota in Neuroendocrine Regulation of Carbohydrate and Lipid Metabolism via the Microbiota-Gut-Brain-Liver Axis. Microorganisms.

[80] Ringseis R., Gessner D.K., Eder K. (2020). The Gut-Liver Axis in the Control of Energy Metabolism and Food Intake in Animals. Annu Rev. Anim. Biosci..

[81] Qin J., Li R., Raes J., Arumugam M., Burgdorf K.S., Manichanh C., Nielsen T., Pons N., Levenez F., Yamada T. (2010). A human gut microbial gene catalogue established by metagenomic sequencing. Nature.

[82] Sender R., Fuchs S., Milo R. (2016). Are We Really Vastly Outnumbered? Revisiting the Ratio of Bacterial to Host Cells in Humans. Cell.

[83] Kissow H., Hartmann B., Holst J.J., Viby N.-E., Hansen L.r.S., Rosenkilde M.M., Hare K.J., Poulsen S.S. (2012). Glucagon-like peptide-1 (GLP-1) receptor agonism or DPP-4 inhibition does not accelerate neoplasia in carcinogen treated mice. Regul. Pept..

[84] Kissow H., Hartmann B., Holst J.J., Poulsen S.S. (2013). Glucagon-like peptide-1 as a treatment for chemotherapy-induced mucositis. Gut.

[85] Koehler J.A., Baggio L.L., Yusta B., Longuet C., Rowland K.J., Cao X., Holland D., Brubaker P.L., Drucker D.J. (2015). GLP-1R agonists promote normal and neoplastic intestinal growth through mechanisms requiring Fgf7. Cell Metab..

[86] Bang-Berthelsen C.H., Holm T.L., Pyke C., Simonsen L., SÃ¸kilde R., Pociot F., Heller R.S., Folkersen L., Kvist P.H., Jackerott M. (2016). GLP-1 induces barrier protective expression in Brunner’s glands and regulates colonic inflammation. Inflamm. Bow. Dis..

[87] Nozu T., Miyagishi S., Kumei S., Nozu R., Takakusaki K., Okumura T. (2018). Glucagone-like peptide-1 analog, liraglutide, improves visceral sensation and gut permeability in rats. J. Gastroenterol. Hepatol..

[88] Hunt J.E., Holst J.J., Jeppesen P.B., Kissow H. (2021). GLP-1 and intestinal diseases. Biomedicines.

[89] Panaro B.L., Yusta B., Matthews D., Koehler J.A., Song Y., Sandoval D.A., Drucker D.J. (2020). Intestine-selective reduction of Gcg expression reveals the importance of the distal gut for GLP-1 secretion. Mol. Metab..

[90] Lu V.B., Rievaj J., Oâ€™Flaherty E.A., Smith C.A., Pais R., Pattison L.A., Tolhurst G., Leiter A.B., Bulmer D.C., Gribble F.M. (2019). Adenosine triphosphate is co-secreted with glucagon-like peptide-1 to modulate intestinal enterocytes and afferent neurons. Nat. Commun..

[91] Osinski C., Le Gléau L., Poitou C., de Toro-Martin J., Genser L., Fradet M., Soula H.A., Leturque A., Blugeon C., Jourdren L. (2021). Type 2 diabetes is associated with impaired jejunal enteroendocrine GLP-1 cell lineage in human obesity. Int. J. Obes..

[92] Harpain F., Schlager L., Hütterer E., Dawoud C., Kirchnawy S., Stift J., Krotka P., Stift A. (2022). Teduglutide in short bowel syndrome patients: A way back to normal life?. J. Parenter. Enter. Nutr..

[93] Bremholm L., Hornum M., Henriksen B.M., Larsen S., Holst J.J. (2009). Glucagon-like peptide-2 increases mesenteric blood flow in humans. Scand. J. Gastroenterol..

[94] Glerup P., Sonne K., Berner-Hansen M., Skarbaliene J. (2022). Short-versus long-term, gender and species differences in the intestinotrophic effects of long-acting glucagon-like peptide 2 analog. Physiol. Res..

[95] Zhang Q., Liu M., Li S., Xu Z., Wang J., Wang Y., Fei Z., Huang W., Sun H. (2018). Oral Bifidobacterium longum expressing GLP-2 improves nutrient assimilation and nutritional homeostasis in mice. J. Microbiol. Methods.

[96] Feng Y., Demehri F.R., Xiao W., Tsai Y.-H., Jones J.C., Brindley C.D., Threadgill D.W., Holst J.J., Hartmann B., Barrett T.A. (2017). Interdependency of EGF and GLP-2 signaling in attenuating mucosal atrophy in a mouse model of parenteral nutrition. Cell. Mol. Gastroenterol. Hepatol..

[97] Kim E.S., Keam S.J. (2017). Teduglutide: A review in short bowel syndrome. Drugs.

[98] Kounatidis D., Vallianou N.G., Tsilingiris D., Christodoulatos G.S., Geladari E., Stratigou T., Karampela I., Dalamaga M. (2022). Therapeutic Potential of GLP-2 Analogs in Gastrointestinal Disorders: Current Knowledge, Nutritional Aspects, and Future Perspectives. Curr. Nutr. Rep..

[99] Brandtzaeg P., Kiyono H., Pabst R., Russell M.W. (2008). Terminology: Nomenclature of mucosa-associated lymphoid tissue. Mucosal Immunol..

[100] Moens E., Veldhoen M. (2011). Epithelial barrier biology: Good fences make good neighbours. Immunology.

[101] McGhee J.R., Fujihashi K. (2012). Inside the mucosal immune system. PLoS Biol..

[102] Zietek T., Rath E. (2016). Inflammation meets metabolic disease: Gut feeling mediated by GLP-1. Front. Immunol..

[103] Arakawa M., Mita T., Azuma K., Ebato C., Goto H., Nomiyama T., Fujitani Y., Hirose T., Kawamori R., Watada H. (2010). Inhibition of monocyte adhesion to endothelial cells and attenuation of atherosclerotic lesion by a glucagon-like peptide-1 receptor agonist, exendin-4. Diabetes.

[104] Al-Dwairi A., Alqudah T.E., Al-Shboul O., Alqudah M., Mustafa A.G., Alfaqih M.A. (2018). Glucagon-like peptide-1 exerts anti-inflammatory effects on mouse colon smooth muscle cells through the cyclic adenosine monophosphate/nuclear factor-ÎºB pathway in vitro. J. Inflamm. Res..

[105] He S., Kahles F., Rattik S., Nairz M., McAlpine C.S., Anzai A., Selgrade D., Fenn A.M., Chan C.T., Mindur J.E. (2019). Gut intraepithelial T cells calibrate metabolism and accelerate cardiovascular disease. Nature.

[106] Tsalamandris S., Antonopoulos A.S., Oikonomou E., Papamikroulis G.-A., Vogiatzi G., Papaioannou S., Deftereos S., Tousoulis D. (2018). The role of inflammation in diabetes: Current concepts and future perspectives. Eur. Cardiol. Rev..

[107] Ramesh P., Yeo J.L., Brady E.M., McCann G.P. (2022). Role of inflammation in diabetic cardiomyopathy. Ther. Adv. Endocrinol. Metab..

[108] Lopez-Candales A., Burgos P.M.H.n., Hernandez-Suarez D.F., Harris D. (2017). Linking chronic inflammation with cardiovascular disease: From normal aging to the metabolic syndrome. J. Nat. Sci..

[109] Lebrun L.J., Lenaerts K., Kiers D., de Barros J.-P.P., Le Guern N., Plesnik J., Thomas C., Bourgeois T., Dejong C.H.C., Kox M. (2017). Enteroendocrine L cells sense LPS after gut barrier injury to enhance GLP-1 secretion. Cell Rep..

[110] Ellingsgaard H., Hauselmann I., Schuler B., Habib A.M., Baggio L.L., Meier D.T., Eppler E., Bouzakri K., Wueest S., Muller Y.D. (2011). Interleukin-6 enhances insulin secretion by increasing glucagon-like peptide-1 secretion from L cells and alpha cells. Nat. Med..

[111] Nguyen A.T., Mandard S., Dray C., Deckert V., Valet P., Besnard P., Drucker D.J., Lagrost L., Grober J. (2014). Lipopolysaccharides-mediated increase in glucose-stimulated insulin secretion: Involvement of the GLP-1 pathway. Diabetes.

[112] Lee Y.S., Park M.S., Choung J.S., Kim S.S., Oh H.H., Choi C.S., Ha S.Y., Kang Y., Kim Y., Jun H.S. (2012). Glucagon-like peptide-1 inhibits adipose tissue macrophage infiltration and inflammation in an obese mouse model of diabetes. Diabetologia.

[113] Bartz S., Mody A., Hornik C., Bain J., Muehlbauer M., Kiyimba T., Kiboneka E., Stevens R., Bartlett J., St Peter J.V. (2014). Severe acute malnutrition in childhood: Hormonal and metabolic status at presentation, response to treatment, and predictors of mortality. J. Clin. Endocrinol. Metab..

[114] Robinson E., Cassidy R.S., Tate M., Zhao Y., Lockhart S., Calderwood D., Church R., McGahon M.K., Brazil D.P., McDermott B.J. (2015). Exendin-4 protects against post-myocardial infarction remodelling via specific actions on inflammation and the extracellular matrix. Basic Res. Cardiol..

[115] Buldak L., Machnik G., Buldak R.J., Labuzek K., Boldys A., Belowski D., Basiak M., Okopień B. (2016). Exenatide (a GLP-1 agonist) expresses anti-inflammatory properties in cultured human monocytes/macrophages in a protein kinase A and B/Akt manner. Pharmacol. Rep..

[116] Guo C., Huang T., Chen A., Chen X., Wang L., Shen F., Gu X. (2016). Glucagon-like peptide 1 improves insulin resistance in vitro through anti-inflammation of macrophages. Braz. J. Med. Biol. Res..

[117] Mitchell P.D., Salter B.M., Oliveria J.-P., El-Gammal A., Tworek D., Smith S.G., Sehmi R., Gauvreau G.M., Butler M., O’Byrne P.M. (2017). Glucagon-like peptide-1 receptor expression on human eosinophils and its regulation of eosinophil activation. Clin. Exp. Allergy.

[118] Steven S., Jurk K., Kopp M., Kröller-Schön S., Mikhed Y., Schwierczek K., Roohani S., Kashani F., Oelze M., Klein T. (2016). Glucagon-like peptide-1 receptor signalling reduces microvascular thrombosis, nitro-oxidative stress and platelet activation in endotoxaemic mice. Br. J. Pharmacol..

[119] Furukawa S., Fujita T., Shimabukuro M., Iwaki M., Yamada Y., Nakajima Y., Nakayama O., Makishima M., Matsuda M., Shimomura I. (2004). Increased oxidative stress in obesity and its impact on metabolic syndrome. J. Clin. Investig..

[120] Dandona P., Aljada A., Bandyopadhyay A. (2004). Inflammation: The link between insulin resistance, obesity and diabetes. Trends Immunol..

[121] Pang J., Feng J.N., Ling W., Jin T. (2022). The anti-inflammatory feature of glucagon-like peptide-1 and its based diabetes drugs-Therapeutic potential exploration in lung injury. Acta Pharm. Sin. B.

[122] Viswanathan P., Chaudhuri A., Bhatia R., Al-Atrash F., Mohanty P., Dandona P. (2007). Exenatide therapy in obese patients with type 2 diabetes mellitus treated with insulin. Endocr. Pract..

[123] Wu J.-D., Xu X.-H., Zhu J., Ding B., Du T.-X., Gao G., Mao X.-M., Ye L., Lee K.-O., Ma J.-H. (2011). Effect of exenatide on inflammatory and oxidative stress markers in patients with type 2 diabetes mellitus. Diabetes Technol. Ther..

[124] Chaudhuri A., Ghanim H., Vora M., Sia C.L., Korzeniewski K., Dhindsa S., Makdissi A., Dandona P. (2012). Exenatide exerts a potent antiinflammatory effect. J. Clin. Endocrinol. Metab..

[125] Hogan A.E., Gaoatswe G., Lynch L., Corrigan M.A., Woods C., O’Connell J., O’Shea D. (2012). Glucagon-like peptide 1 analogue therapy directly modulates innate immune-mediated inflammation in individuals with type 2 diabetes mellitus. Diabetologia.

[126] Zatorski H., Salaga M., Fichna J. (2019). Role of glucagon-like peptides in inflammatory bowel diseasesâ€”current knowledge and future perspectives. Naunyn-Schmiedeberg’s Arch. Pharmacol..

[127] Anbazhagan A.N., Thaqi M., Priyamvada S., Jayawardena D., Kumar A., Gujral T., Chatterjee I., Mugarza E., Saksena S., Onyuksel H. (2017). GLP-1 nanomedicine alleviates gut inflammation. Nanomed. Nanotechnol. Biol. Med..

[128] Lourie J. (2019). A Novel Use of Liraglutide: Induction of Partial Remission in Ulcerative Colitis and Ankylosing Spondylitis. Clin. Med. Rev. Case Rep.

[129] Robertson M.D., Livesey G., Morgan L.M., Hampton S.M., Mathers J.C. (1999). The influence of the colon on postprandial glucagon-like peptide 1 (7-36) amide concentration in man. J. Endocrinol..

[130] Keller J., Binnewies U., RÃ¶sch M., Juul Holst J., Beglinger C., Andresen V., Layer P. (2015). Gastric emptying and disease activity in inflammatory bowel disease. Eur. J. Clin. Investig..

[131] Xie S., Liu B., Fu S., Wang W., Yin Y., Li N., Chen W., Liu J., Liu D. (2014). GLP-2 suppresses LPS-induced inflammation in macrophages by inhibiting ERK phosphorylation and NF-ÎºB activation. Cell. Physiol. Biochem..

[132] Deng G., Lei Q., Gao X., Zhang Y., Zheng H., Bi J., Wang X. (2021). Glucagon-Like Peptide-2 Modulates Enteric Paneth Cells Immune Response and Alleviates Gut Inflammation During Intravenous Fluid Infusion in Mice With a Central Catheter. Front. Nutr..

[133] Wang C., Fan W., Feng X., Zhang Y., Liu C., Liu Z. (2021). The roles of the glucagon-like peptide-2 and the serum TGF-Î²1 levels in the intestinal barrier and immune functions in rats with obstructive jaundice. Am. J. Transl. Res..

[134] Martin G.R., Wallace L.E., Sigalet D.L. (2004). Glucagon-like peptide-2 induces intestinal adaptation in parenterally fed rats with short bowel syndrome. Am. J. Physiol. Gastrointest. Liver Physiol..

[135] Chang Y., Deng Q., Zhang Z., Zhao H., Tang J., Chen X., Liu G., Tian G., Cai J., Jia G. (2021). Glucagon-like peptide 2 attenuates intestinal mucosal barrier injury through the MLCK/pMLC signaling pathway in a piglet model. J. Cell. Physiol..

[136] Norona J., Apostolova P., Schmidt D., Ihlemann R., Reischmann N., Taylor G., Köhler N., de Heer J., Heeg S., Andrieux G. (2020). Glucagon-like peptide 2 for intestinal stem cell and Paneth cell repair during graft-versus-host disease in mice and humans. Blood.

[137] Madnawat H., Welu A.L., Gilbert E.J., Taylor D.B., Jain S., Manithody C., Blomenkamp K., Jain A.K. (2020). Mechanisms of parenteral nutrition–associated liver and gut injury. Nutr. Clin. Pract..

[138] Hellerman Itzhaki M., Singer P. (2020). Advances in medical nutrition therapy: Parenteral nutrition. Nutrients.

[139] Belkaid Y., Hand T.W. (2014). Role of the microbiota in immunity and inflammation. Cell.

[140] Belkaid Y., Harrison O.J. (2017). Homeostatic immunity and the microbiota. Immunity.

[141] Hooper L.V., Littman D.R., Macpherson A.J. (2012). Interactions between the microbiota and the immune system. Science.

[142] Klein J.R. (2004). T-cell activation in the curious world of the intestinal intraepithelial lymphocyte. Immunol. Res..

[143] Kamada N., Seo S.-U., Chen G.Y., Núñez G. (2015). Role of the gut microbiota in immunity and inflammatory disease. Nat. Rev. Immunol..

[144] Francino M.P. (2014). Early development of the gut microbiota and immune health. Pathogens.

[145] Worthington J.J. (2015). The intestinal immunoendocrine axis: Novel cross-talk between enteroendocrine cells and the immune system during infection and inflammatory disease. Biochem. Soc. Trans..

[146] Watnick P.I., Jugder B.-E. (2020). Microbial control of intestinal homeostasis via enteroendocrine cell innate immune signaling. Trends Microbiol..

[147] Roed S.N., Wismann P., Underwood C.R., Kulahin N., Iversen H., Cappelen K.A., Schäffer L., Lehtonen J., Hecksher-Soerensen J., Secher A. (2014). Real-time trafficking and signaling of the glucagon-like peptide-1 receptor. Mol. Cell. Endocrinol..

[148] Clarke G., Stilling R.M., Kennedy P.J., Stanton C., Cryan J.F., Dinan T.G. (2014). Minireview: Gut microbiota: The neglected endocrine organ. Mol. Endocrinol..

[149] Hacquard S., Garrido-Oter R., González A., Spaepen S., Ackermann G., Lebeis S., McHardy A.C., Dangl J.L., Knight R., Ley R. (2015). Microbiota and host nutrition across plant and animal kingdoms. Cell Host Microbe.

[150] Lynch J.B., Hsiao E.Y. (2019). Microbiomes as sources of emergent host phenotypes. Science.

[151] Cani P.D., Van Hul M. (2020). Mediterranean diet, gut microbiota and health: When age and calories do not add up!. Gut.

[152] Xiao H., Kang S. (2020). The Role of the Gut Microbiome in Energy Balance With a Focus on the Gut-Adipose Tissue Axis. Front Genet.

[153] Turnbaugh P.J., Bäckhed F., Fulton L., Gordon J.I. (2008). Diet-induced obesity is linked to marked but reversible alterations in the mouse distal gut microbiome. Cell Host Microbe.

[154] Cahenzli J., Köller Y., Wyss M., Geuking M.B., McCoy K.D. (2013). Intestinal microbial diversity during early-life colonization shapes long-term IgE levels. Cell Host Microbe.

[155] Bauer H., Horowitz R.E., Levenson S.M., Popper H. (1963). The response of the lymphatic tissue to the microbial flora. Studies on germfree mice. Am. J. Pathol..

[156] Herbst T., Sichelstiel A., Schär C., Yadava K., Bürki K., Cahenzli J., McCoy K., Marsland B.J., Harris N.L. (2013). Dysregulation of allergic airway inflammation in the absence of microbial colonization. Am. J. Respir. Crit. Care Med..

[157] Hapfelmeier S., Lawson M.A.E., Slack E., Kirundi J.K., Stoel M., Heikenwalder M., Cahenzli J., Velykoredko Y., Balmer M.L., Endt K. (2010). Reversible microbial colonization of germ-free mice reveals the dynamics of IgA immune responses. Science.

[158] Umesaki Y., Setoyama H., Matsumoto S., Okada Y. (1993). Expansion of alpha beta T-cell receptor-bearing intestinal intraepithelial lymphocytes after microbial colonization in germ-free mice and its independence from thymus. Immunology.

[159] Wells J.M., Rossi O., Meijerink M., van Baarlen P. (2011). Epithelial crosstalk at the microbiota–mucosal interface. Proc. Natl. Acad. Sci. USA.

[160] Tan T.G., Sefik E., Geva-Zatorsky N., Kua L., Naskar D., Teng F., Pasman L., Ortiz-Lopez A., Jupp R., Wu H.-J.J. (2016). Identifying species of symbiont bacteria from the human gut that, alone, can induce intestinal Th17 cells in mice. Proc. Natl. Acad. Sci. USA.

[161] Olszak T., An D., Zeissig S., Vera M.P., Richter J., Franke A., Glickman J.N., Siebert R., Baron R.M., Kasper D.L. (2012). Microbial exposure during early life has persistent effects on natural killer T cell function. Science.

[162] Jiao Y., Wu L., Huntington N.D., Zhang X. (2020). Crosstalk between gut microbiota and innate immunity and its implication in autoimmune diseases. Front. Immunol..

[163] Danne C., Ryzhakov G., Martínez-López M., Ilott N.E., Franchini F., Cuskin F., Lowe E.C., Bullers S.J., Arthur J.S.C., Powrie F. (2017). A large polysaccharide produced by Helicobacter hepaticus induces an anti-inflammatory gene signature in macrophages. Cell Host Microbe.

[164] Bansal T., Alaniz R.C., Wood T.K., Jayaraman A. (2010). The bacterial signal indole increases epithelial-cell tight-junction resistance and attenuates indicators of inflammation. Proc. Natl. Acad. Sci. USA.

[165] Bevins C.L., Salzman N.H. (2011). Paneth cells, antimicrobial peptides and maintenance of intestinal homeostasis. Nat. Rev. Microbiol..

[166] Ehmann D., Wendler J., Koeninger L., Larsen I.S., Klag T., Berger J., Marette A., Schaller M., Stange E.F., Malek N.P. (2019). Paneth cell Î±-defensins HD-5 and HD-6 display differential degradation into active antimicrobial fragments. Proc. Natl. Acad. Sci. USA.

[167] Zheng Y., Wu Y., Tao L., Chen X., Jones T.J., Wang K., Hu F. (2020). Chinese Propolis Prevents Obesity and Metabolism Syndromes Induced by a High Fat Diet and Accompanied by an Altered Gut Microbiota Structure in Mice. Nutrients.

[168] Peterson D.A., McNulty N.P., Guruge J.L., Gordon J.I. (2007). IgA response to symbiotic bacteria as a mediator of gut homeostasis. Cell Host Microbe.

[169] Benckert J., Schmolka N., Kreschel C., Zoller M.J., Sturm A., Wiedenmann B., Wardemann H. (2011). The majority of intestinal IgA+ and IgG+ plasmablasts in the human gut are antigen-specific. J. Clin. Investig..

[170] Janeway Jr C.A., Medzhitov R. (2002). Innate immune recognition. Annu. Rev. Immunol..

[171] Rakoff-Nahoum S., Paglino J., Eslami-Varzaneh F., Edberg S., Medzhitov R. (2004). Recognition of commensal microflora by toll-like receptors is required for intestinal homeostasis. Cell.

[172] Carvalho F.A., Koren O., Goodrich J.K., Johansson M.E.V., Nalbantoglu I., Aitken J.D., Su Y., Chassaing B., Walters W.A., González A. (2012). Transient inability to manage proteobacteria promotes chronic gut inflammation in TLR5-deficient mice. Cell Host Microbe.

[173] Vijay-Kumar M., Aitken J.D., Carvalho F.A., Cullender T.C., Mwangi S., Srinivasan S., Sitaraman S.V., Knight R., Ley R.E., Gewirtz A.T. (2010). Metabolic syndrome and altered gut microbiota in mice lacking Toll-like receptor 5. Science.

[174] Furusawa Y., Obata Y., Fukuda S., Endo T.A., Nakato G., Takahashi D., Nakanishi Y., Uetake C., Kato K., Kato T. (2013). Commensal microbe-derived butyrate induces the differentiation of colonic regulatory T cells. Nature.

[175] Yadav H., Lee J.H., Lloyd J., Walter P., Rane S.G. (2013). Beneficial metabolic effects of a probiotic via butyrate-induced GLP-1 hormone secretion. J. Biol. Chem..

[176] Schulthess J., Pandey S., Capitani M., Rue-Albrecht K.C., Arnold I., Franchini F., Chomka A., Ilott N.E., Johnston D.G.W., Pires E. (2019). The short chain fatty acid butyrate imprints an antimicrobial program in macrophages. Immunity.

[177] Canfora E.E., Jocken J.W., Blaak E.E. (2015). Short-chain fatty acids in control of body weight and insulin sensitivity. Nat. Rev. Endocrinol..

[178] Wu K., Yuan Y., Yu H., Dai X., Wang S., Sun Z., Wang F., Fei H., Lin Q., Jiang H. (2020). The gut microbial metabolite trimethylamine N-oxide aggravates GVHD by inducing M1 macrophage polarization in mice. Blood.

[179] Walker A.W., Sanderson J.D., Churcher C., Parkes G.C., Hudspith B.N., Rayment N., Brostoff J., Parkhill J., Dougan G., Petrovska L. (2012). High-throughput clone library analysis of the mucosa-associated microbiota reveals dysbiosis and differences between inflamed and non-inflamed regions of the intestine in inflammatory bowel disease. BMC Microbiol..

[180] Wang Z.-K., Yang Y.-S., Chen Y., Yuan J., Sun G., Peng L.-H. (2014). Intestinal microbiota pathogenesis and fecal microbiota transplantation for inflammatory bowel disease. World J. Gastroenterol. WJG.

[181] Vijay A., Valdes A.M. (2021). Role of the gut microbiome in chronic diseases: A narrative review. Eur. J. Clin. Nutr..

[182] Covasa M., Stephens R.W., Toderean R., Cobuz C. (2019). Intestinal sensing by gut microbiota: Targeting gut peptides. Front. Endocrinol..

[183] Psichas A., Sleeth M.L., Murphy K.G., Brooks L., Bewick G.A., Hanyaloglu A.C., Ghatei M.A., Bloom S.R., Frost G. (2015). The short chain fatty acid propionate stimulates GLP-1 and PYY secretion via free fatty acid receptor 2 in rodents. Int. J. Obes. (Lond.).

[184] Tolhurst G., Heffron H., Lam Y.S., Parker H.E., Habib A.M., Diakogiannaki E., Cameron J., Grosse J., Reimann F., Gribble F.M. (2012). Short-chain fatty acids stimulate glucagon-like peptide-1 secretion via the G-protein-coupled receptor FFAR2. Diabetes.

[185] Li Y., Kokrashvili Z., Mosinger B., Margolskee R.F. (2013). Gustducin couples fatty acid receptors to GLP-1 release in colon. Am. J. Physiol. Endocrinol. Metab..

[186] Christiansen C.B., Gabe M.B.N., Svendsen B., Dragsted L.O., Rosenkilde M.M., Holst J.J. (2018). The impact of short-chain fatty acids on GLP-1 and PYY secretion from the isolated perfused rat colon. Am. J. Physiol. Gastrointest. Liver Physiol..

[187] Chimerel C., Emery E., Summers D.K., Keyser U., Gribble F.M., Reimann F. (2014). Bacterial metabolite indole modulates incretin secretion from intestinal enteroendocrine L cells. Cell Rep..

[188] Wichmann A., Allahyar A., Greiner T.U., Plovier H., Lundén G.Ö., Larsson T., Drucker D.J., Delzenne N.M., Cani P.D., Bäckhed F. (2013). Microbial modulation of energy availability in the colon regulates intestinal transit. Cell Host Microbe.

[189] Brooks L., Viardot A., Tsakmaki A., Stolarczyk E., Howard J.K., Cani P.D., Everard A., Sleeth M.L., Psichas A., Anastasovskaj J. (2017). Fermentable carbohydrate stimulates FFAR2-dependent colonic PYY cell expansion to increase satiety. Mol. Metab..

[190] Zhao L., Chen Y., Xia F., Abudukerimu B., Zhang W., Guo Y., Wang N., Lu Y. (2018). A glucagon-like peptide-1 receptor agonist lowers weight by modulating the structure of gut microbiota. Front. Endocrinol..

[191] Madsen M.S.A., Holm J.B., Pallejà A., Wismann P., Fabricius K., Rigbolt K., Mikkelsen M., Sommer M., Jelsing J., Nielsen H.B. (2019). Metabolic and gut microbiome changes following GLP-1 or dual GLP-1/GLP-2 receptor agonist treatment in diet-induced obese mice. Sci. Rep..

[192] Grasset E., Puel A., Charpentier J., Collet X., Christensen J.E., TercÃ© F.o., Burcelin R.m. (2017). A specific gut microbiota dysbiosis of type 2 diabetic mice induces GLP-1 resistance through an enteric NO-dependent and gut-brain axis mechanism. Cell Metab..

[193] He Y., Wu W., Zheng H.-M., Li P., McDonald D., Sheng H.-F., Chen M.-X., Chen Z.-H., Ji G.-Y., Zheng Z.-D.-X. (2018). Regional variation limits applications of healthy gut microbiome reference ranges and disease models. Nat. Med..

[194] Wang Z., Saha S., Van Horn S., Thomas E., Traini C., Sathe G., Rajpal D.K., Brown J.R. (2018). Gut microbiome differences between metformin-and liraglutide-treated T2 DM subjects. Endocrinol. Diabetes Metab..

[195] Shang J., Liu F., Zhang B., Dong K., Lu M., Jiang R., Xu Y., Diao L., Zhao J., Tang H. (2021). Liraglutide-induced structural modulation of the gut microbiota in patients with type 2 diabetes mellitus. PeerJ.

[196] Montandon S.A., Jornayvaz F.o.R. (2017). Effects of antidiabetic drugs on gut microbiota composition. Genes.

[197] Wang L., Li P., Tang Z., Yan X., Feng B. (2016). Structural modulation of the gut microbiota and the relationship with body weight: Compared evaluation of liraglutide and saxagliptin treatment. Sci. Rep..

[198] Smits M.M., Tonneijck L., Muskiet M.H.A., Hoekstra T., Kramer M.H.H., Diamant M., Nieuwdorp M., Groen A.K., Cahen D.L., van Raalte D.l.H. (2016). Biliary effects of liraglutide and sitagliptin, a 12-week randomized placebo-controlled trial in type 2 diabetes patients. Diabetes Obes. Metab..

[199] Everard A., Belzer C., Geurts L., Ouwerkerk J.P., Druart C.l., Bindels L.B., Guiot Y., Derrien M., Muccioli G.G., Delzenne N.M. (2016). Cross-talk between Akkermansia muciniphila and intestinal epithelium controls diet-induced obesity. Proc. Natl. Acad. Sci. USA.

[200] Yan M., Song M.-M., Bai R.-X., Cheng S., Yan W.-M. (2016). Effect of Roux-en-Y gastric bypass surgery on intestinal Akkermansia muciniphila. World J. Gastrointest. Surg..

[201] Dao M.C., Everard A., Aron-Wisnewsky J., Sokolovska N., Prifti E., Verger E.O., Kayser B.D., Levenez F., Chilloux J., Hoyles L. (2016). Akkermansia muciniphila and improved metabolic health during a dietary intervention in obesity: Relationship with gut microbiome richness and ecology. Gut.

[202] Tsai C.-Y., Lu H.-C., Chou Y.-H., Liu P.-Y., Chen H.-Y., Huang M.-C., Lin C.-H., Tsai C.-N. (2022). Gut Microbial Signatures for Glycemic Responses of GLP-1 Receptor Agonists in Type 2 Diabetic Patients: A Pilot Study. Front. Endocrinol..

[203] De Vadder F., Kovatcheva-Datchary P., Goncalves D., Vinera J., Zitoun C., Duchampt A., Backhed F., Mithieux G. (2014). Microbiota-generated metabolites promote metabolic benefits via gut-brain neural circuits. Cell.

[204] Wahlstrom A., Sayin S.I., Marschall H.U., Backhed F. (2016). Intestinal Crosstalk between Bile Acids and Microbiota and Its Impact on Host Metabolism. Cell Metab..

[205] Dalile B., Van Oudenhove L., Vervliet B., Verbeke K. (2019). The role of short-chain fatty acids in microbiota-gut-brain communication. Nat. Rev. Gastroenterol. Hepatol..

[206] Silva Y.P., Bernardi A., Frozza R.L. (2020). The Role of Short-Chain Fatty Acids From Gut Microbiota in Gut-Brain Communication. Front. Endocrinol..

[207] Cani P.D., Possemiers S., Van de Wiele T., Guiot Y., Everard A., Rottier O., Geurts L., Naslain D., Neyrinck A., Lambert D.M. (2009). Changes in gut microbiota control inflammation in obese mice through a mechanism involving GLP-2-driven improvement of gut permeability. Gut.

[208] Li D., Yang Y., Yin X., Liu Y., Xu H., Ni Y., Hang P., Niu S., Zhang H., Ding W. (2021). Glucagon-like peptide (GLP)-2 improved colonizing bacteria and reduced severity of ulcerative colitis by enhancing the diversity and abundance of intestinal mucosa. Bioengineered.

[209] Wu J., Ren W., Li L., Luo M., Xu K., Shen J., Wang J., Chang G., Lu Y., Qi Y. (2018). Effect of aging and glucagon-like peptide 2 on intestinal microbiota in SD rats. Aging Dis..

[210] Simon M.-C., Strassburger K., Nowotny B., Kolb H., Nowotny P., Burkart V., Zivehe F., Hwang J.-H., Stehle P., Pacini G. (2015). Intake of Lactobacillus reuteri improves incretin and insulin secretion in glucose-tolerant humans: A proof of concept. Diabetes Care.

